# Mapping Global Research on Depression Among Medical Students: A Bibliometric and Conceptual Analysis (2001–2025)

**DOI:** 10.21315/mjms-02-2026-125

**Published:** 2026-08-31

**Authors:** Ayesha Yasin, Nabisah Ibrahim

**Affiliations:** School of Applied Psychology, Social Work and Policy (SAPSP), Universiti Utara Malaysia, 06010 UUM, Sintok, Kedah, Malaysia

**Keywords:** medical students, depression, global, bibliometric analysis, conceptual framework, research trends, mental health

## Abstract

**Background:**

The importance of mental health among medical students has gained worldwide recognition, especially after the COVID-19 pandemic in 2019. High rates of depression among medical students have drawn the attention of researchers, who have sought to identify and explore the factors related to the onset of depression. This study conducted a comprehensive review of the literature on depression among medical students and developed a conceptual framework highlighting the interaction of various factors contributing to the onset and maintenance of depression.

**Methods:**

This study conducted a bibliometric analysis of 467 papers retrieved from the Scopus database between 2001 and 2025. The eligibility criterion was articles that contained the terms “depression” and “medical students” in their titles. This systematic approach ensured a manageable dataset for analysis. Microsoft Excel and VOSviewer were used for an in-depth analysis of publication trends, affiliations, countries, prolific authors, and major journals and sources regarding depression among medical students.

**Results:**

The highest number of papers published was in 2022, while the United States was identified as the most productive country. Co-occurrence analysis identified eight core themes: psychosocial factors, affective–cognitive factors, psychological stressors, coping mechanisms, burnout, mental health and well-being, academic performance, and lifestyle factors of depression among medical students.

**Conclusion:**

This study significantly contributes to the field by constructing a comprehensive conceptual framework that logically organises thematic factors into predictors, moderators, and outcome variables, and is expected to guide future research and align with emerging trends in this domain. Additionally, this study highlights the need for global collaboration on many topics in this field and urges the development of suitable psychological preventive strategies to reduce the rate of depression among medical students.

## Introduction

Depression is one of the most common mental health disorders. A global mental health concern for a decade, it is characterised by persistent sadness, loss of interest, and a range of cognitive and physical symptoms that interfere with daily functioning (1). The average onset of depression has been found to occur in those aged 21 to 25 years, which is also the transition period from adolescence to adulthood. Young adults, particularly those in higher education, are thus at increased risk of developing depression. Several studies have reported that depression is more prevalent among medical students than the general student population. This heightened vulnerability is attributed to the psychosocial stressors and developmental transitions of this stage of life (2). Additionally, the unique pressures and challenges of medical training, including high academic competition, long study hours, emotional exhaustion, and lack of extracurricular activities, further exacerbate the risk of depression (3, 4). Moreover, the reported global prevalence of depression varies by region and methodology (5).

Despite extensive research on depression among medical students, several gaps in our knowledge remain. First, there is limited comparability across studies due to the heterogeneity of the assessment measures and varying cutoff scores used (4). Second, there is an under-reporting of certain factors due to social stigma (6). Third, because of a lack of longitudinal studies, there is limited understanding of trends over time. Moreover, as the majority of the research is concentrated in high-income countries, this has created a gap in our understanding of the effects of socioeconomic and systemic educational factors in low-income countries (5).

Accordingly, this bibliometric analysis examined global research output on depression among medical students using the Scopus database. For this study, medical students are defined as individuals enrolled in undergraduate or graduate medical programmes (MBBS, MD, or equivalent), and depression encompasses both clinical depression (e.g., major depressive disorder) and depressive symptoms as assessed by standardised instruments such as the PHQ-9, DASS-21, BDI-II, and CES-D.

Given the high prevalence of depression among medical students, it is critical to understand the scope and direction of research in this field. Although several reviews have examined the prevalence and risk factors of depression among medical students (4, 7), no bibliometric research has been conducted to comprehensively map the evolution of this research domain, including thematic hot spots and knowledge gaps, via a bibliometric approach to systematically assess the volume, impact, and development of scholarly output across time and regions. This study seeks to answer the following research questions (RQ):

- RQ1: What are the major keywords used in research on depression among medical students?
- RQ2: What emerging themes can be identified from the literature?
- RQ3: What are the future directions of the emerging themes?

Bibliometric analysis is a powerful method for identifying current trends in a field of research (8, 9) that enables researchers to examine the thematic focus of recurring keywords and patterns in the data (10). Therefore, in this study, publications from 2000 to July 2025 were collected from the Scopus database, one of the largest and most comprehensive citation databases (11). The analysis was conducted using VOSviewer (version 1.6.15) (12).

This bibliometric analysis systematically identifies research gaps and trends to provide guidance for researchers, educators, medical curriculum planners, and mental health policymakers. By identifying key contributors, trending topics, and under-researched areas, the findings can inform targeted interventions, support global collaborations, and shape funding priorities. For medical institutions, the results highlight the importance of mental health support systems and evidence-informed curriculum reforms. For governments and global health organisations, the data underscore the need for structural changes and mental health policies tailored to medical student populations.

## Methods

Bibliometric analysis is a quantitative method for mapping the research landscape of a specific topic. It can reveal trends, thematic concentrations, and evolving patterns within a field, yielding insights into topic development and potential future directions (13, 14). It provides statistical outputs that approximate the growth rates, topic saturation, and thematic concentration within a field. This study employed bibliometric analysis to explore the global literature on depression among medical students, with a particular focus on keywords and emerging themes. The dataset used in this study was extracted from the Scopus database, chosen for its coverage across disciplines and flexible search options (15).

Tools such as VOSviewer (version 1.6.15) were employed to visualise and analyse keyword co-occurrences and emerging thematic patterns. This approach directly addresses the study’s research questions regarding the major keywords and emerging themes in depression research among medical students (16). Figure 1 provides a PRISMA-based flowchart of the article screening and selection process. Despite the use of a carefully constructed search string, some retrieved articles fell outside the scope of this study and were excluded during the title and abstract screening.

### Data Source and Search Strings

The dataset consists of articles retrieved from the Scopus database. The Scopus database was used because of its extensive coverage of peer-reviewed literature across the health and social sciences. The Scopus database is also highly compatible with VOSviewer. The literature search was conducted on 28 July 2025, using the keywords “depression,” “major depression,” “depressive symptoms,” “medical students,” and “undergraduate medical students” with the appropriate Boolean operators (OR, AND). The search was limited to the titles, abstracts, and keywords of each article. The search query strings used included TITLE-ABS-KEY (“depression” OR “major depression” OR “depressive symptom*”) AND (“medical student*” OR “undergraduate medical student”). The initial search yielded 4,500 articles.

#### Data Screening

The retrieved records were refined with predefined inclusion criteria to ensure the relevance and quality of the study. First, the records were limited to publications published between 2000 and 2025. Second, the records were limited to articles published in English. Third, only records with a final publication status were included. Fourth, peer-reviewed articles were included; editorials, conference papers, book chapters, and other document types were excluded. Fifth, the search was defined using relevant keywords such as medical students and depression. These inclusion criteria reduced the number of articles to 963.

The articles were further screened by title and abstract by the primary researcher, who assessed the eligibility of the studies, excluding studies conducted on populations other than medical students or whose primary outcome was something other than depression. This process screened out another 487 articles, leaving 476 eligible articles for further analysis.

### Eligibility Criteria

The following eligibility criteria were followed to ensure methodological rigour and thematic reverence.

#### Inclusion Criteria

The articles were included if they were:

- Published between January 2000 and July 2025
- Indexed in the Scopus database
- At the final stage of publication
- Focused specifically on depression among medical students

#### Exclusion Criteria

Articles were excluded if:

- They focused on a population other than medical students
- Depression was not the primary outcome
- They were conference papers, commentaries, letters, or editorials.

### Data Analysis

After screening, a total of 476 documents were included in the final bibliometric analysis. VOSviewer (version 1.6.20) was employed to perform bibliometric analysis by visualising and analysing the relationships within datasets. Author keywords were used to conduct a keyword co-occurrence analysis to identify major themes and trends in the literature, as keywords represent the core conceptual content of published research and provide insight into dominant and emerging areas within the field. Only keywords meeting a minimum threshold of five occurrences were included in the network visualisation. Related keywords based on the co-occurrence patterns were grouped by the VOSviewer algorithm.

Keywords were standardised to ensure consistency in the co-occurrence network, and synonymous terms, plural forms, and variations in spelling were merged into unified keywords. For example, the keywords “depressive symptoms,” “depressive disorder,” and “depression” were merged into one standard keyword: “depression.” Similarly, variations in keywords such as “medical student” and “undergraduate students, medical” were merged into “medical students.” Keywords related to country names and assessment measures were excluded to maintain the focus on the conceptual relationships among the risk factors associated with depression. The standardisation process was conducted using a combination of the VOSviewer thesaurus file and manual data cleaning to enhance the accuracy and interpretability of the network visualisation.

## Results

The results emphasise keyword co-occurrences, networks, and thematic clustering, which form the basis for identifying emerging themes and future research directions. The results of the bibliometric analysis are discussed below.

### Overview of the Dataset

The dataset includes 476 articles, which comprise 363 original articles, followed by 55 systematic and meta-analysis articles, 30 experimental studies, 12 mixed-method studies, and a smaller group of eight qualitative studies, five scoping reviews, and three short reports.

### Keyword Frequency Analysis

Keyword co-occurrence analysis was conducted to identify prominent themes in studies of depression among medical students. A total of 699 keywords were extracted from the articles in the database, 45 of which had counts exceeding the threshold value and were included in the analysis. The main keywords were “depression” (links 33; 325 occurrences) and “medical students” (links 32; 225 occurrences), indicating their central role within the research network. Other commonly occurring keywords included anxiety, stress, psychological stress, and burnout, reflecting the multidimensional nature of depression research in medical education. Keyword frequency was visually indicated by the font size and larger nodes in the network representation (17).

### Keyword Co-occurrence Network

A keyword co-occurrence network was generated to examine the relationships among frequently used keywords and identify research clusters within the literature. In the network visualisation, the strengths of the correlations among the keywords were indicated by the thickness of the lines connecting the keywords (18), while cluster membership was indicated by the same colour. The co-occurrence analysis yielded eight distinct clusters comprising 190 links among selected keywords, indicated in different colours in Figure 2. These clusters represent groups of keywords that frequently appear together, indicating shared thematic and conceptual orientation within the literature. The strong interconnections between central keywords such as depression, medical students, anxiety, and stress indicate that research in this domain is highly interconnected, with overlapping psychological, academic, and contextual dimensions.

### Identification of Themes and Cluster Formation

The themes were identified through systematic keyword co-occurrence analysis using VOSviewer. The steps in identifying the themes and cluster formation are shown in Figure 3. As noted above, 476 documents were uploaded to VOSviewer, and 699 author keywords were extracted. Only author keywords were used in this study for the co-occurrence analysis, as they reflect the conceptual and thematic orientation of the author (13) and are thus more precise than indexed keywords in identifying emerging themes and evolving terminology within the research domain; index keywords are assigned by algorithms, and while they provide standardised terminology, they fail to capture the conceptual meaning intended by the author.

The analytical robustness of the most frequently used terms was enhanced in the second step through the omission of keywords falling below the threshold, as discussed earlier.

A keyword co-occurrence network based on frequency and link strengths between the terms was then generated using VOSviewer’s clustering algorithm system, which is based on the association strengths between terms.

The algorithm identified eight different clusters, each depicted in a different colour in the network visualisation, as shown in Figure 2. Keywords in the same cluster display higher internal connectivity and thematic similarity than they do with keywords in other clusters. The thematic labelling of the clusters was performed through interpretive synthesis as follows: The most frequently occurring keywords within each cluster were examined, and their associations with other keywords within the cluster were analysed. Representative studies related to each cluster were reviewed for the conceptual coherence of the keywords within the same cluster. The thematic labelling represents the conceptual coherence of the keywords and was based on earlier research to ensure theoretical plausibility. The clusters that emerged during the analysis were interpreted as emerging themes and include predictors, moderators, and outcomes associated with depression among medical students.

### Emerging Themes

The eight main themes derived from the clustering in the keyword co-occurrence network represent major research directions in depression among medical students, as follows.

#### Cluster 1: Psychosocial Factors

This cluster includes keywords such as academic stress (links 9, occurrences 7), depressive symptoms (links 20, occurrences 28), gender (links 5, occurrences 5), medical students (links 32, occurrences 225), personality traits (links 8, occurrences 4), social support (links 8, occurrences 7), and suicidal ideation (links 12, occurrences 12) that concern the psychosocial vulnerabilities in depression.

#### Cluster 2: Affective–Cognitive Processes

Cluster 2 includes keywords such as attitude (links 4, occurrences 5), depression (links 33, occurrences 325), emotional regulation (links 6, occurrences 4), empathy (links 7, occurrences 5), insomnia (links 7, occurrences 6), and knowledge (links 4, occurrences 5), reflecting the cognitive and emotional mechanisms associated with depression experiences.

#### Cluster 3: Coping Mechanism

This cluster comprises coping (links 7, occurrences 6), medical education (links 12, occurrences 23), perceived stress (links 8, occurrences 7), resilience (links 11, occurrences 7), smartphone addiction (links 7, occurrences 5), and sleep quality (links 11, occurrences 15), which concern adaptive and maladaptive responses to stress.

#### Cluster 4: Psychological Stressors

This cluster included anxiety (links 27, occurrences 138), internet addiction (links 5, occurrences 8), COVID-19 (links 21, occurrences 49), pandemic (links 8, occurrences 7), psychological distress (links 7, occurrences 5), and stress (links 23, occurrences 63), which are related to the acute and chronic stressors affecting mental health.

#### Cluster 5: Burnout

This cluster includes burnout (links 11, occurrences 17), sleep (links 7, occurrences 6), and suicide (links 5, occurrences 5), which concern exhaustion-related outcomes linked to depressive symptoms.

#### Cluster 6: Mental Health and Well-Being

The keywords in this cluster, such as mental health (links 23, occurrences 52), risk factors (links 5, occurrences 6) and well-being (links 7, occurrences 6), relate to a holistic mental health perspective.

#### Cluster 7: Lifestyle Factors

This cluster included physical activity (links 5, occurrences 6) and quality of life (links 7, occurrences 5), which are related to health-related behaviours associated with depression.

#### Cluster 8: Academic Performance Outcome

The keyword academic performance (links 7, occurrences 9) formed a distinct cluster, reflecting outcome-oriented research linking depression to academic functioning.

## Discussion

### Overview of Research Trends and Thematic Evolution

This analysis indicates that research on depression among medical students comprises multiple interconnected themes, as indicated by the keyword co-occurrence analysis. Since 2000, research on this topic has evolved from the study of general mental health concerns to the exploration of more relevant factors in the disorder, such as coping strategies, psychological stressors, various challenges related to technology use, coping with the pandemic, and certain institutional and environmental factors. The density of keyword clusters in terms of occurrences reflects a growing scholarly interest in conceptualising depression not only as an individual mental health issue but also as a multi-factorial outcome caused by various factors such as academic, technological, lifestyle, and institutional factors.

This thematic mapping of the keywords affords scholars a more structured understanding of the research landscape and highlights the need for further inquiry, particularly regarding factors like resilience, digital dependency, and structural stressors that affect medical students during their training. These findings also point to the need for research that integrates cultural, pedagogical, and policy-level interventions to address the complex causes of depression in medical students. Overall, this study of depression among medical students identified strong research interest in psychological stressors, coping strategies, institutional challenges, digital dependency, and lifestyle factors, as well as a variety of emerging themes such as smartphone addiction, resilience, sleep quality, academic performance, and lifestyle factors.

### Interpretation of Keyword Co-Occurrence and Cluster Structure

Similarly, our keyword co-occurrence mapping analysis revealed trends in the research on depression among medical students. As noted above, the analysis of 45 keywords yielded eight clusters with 188 connections. Among these keywords, the term “depression” had 325 occurrences and 33 links, and “medical students” had 225 occurrences, indicating the interconnected thematic domains in research on depression among medical students and indicating possibilities for further in-depth analysis based on the identified bibliometric clusters.

### Conceptual Synthesis Framework of Depression among Medical Students

Regarding conceptual framework development (RQ2), eight main clusters were found, consisting of a variety of factors that contribute to the onset and maintenance of depression among medical students. Based on this, we propose the conceptual framework and structural linkages shown in Figure 4. This framework is in line with the results of earlier studies, supporting the validity of the proposed framework for the study of depression among medical students (Clusters 1–7).

This conceptual framework is expected to promote a more robust scholarly understanding of research in this field, providing novice researchers with a structured and comprehensive strategy for in-depth analysis and enhancing researchers’ ability to identify the relationships among several risk factors of depression.

The clusters in this bibliometric analysis reflect the multifaceted nature of depression as a disorder: One factor alone does not suffice to explain its onset and maintenance. The number of factors directly related to depression, as well as their interconnections, impacts the onset and persistence of depression in medical students. Of the eight clusters identified in this bibliometric analysis, four (Clusters 1, 2, 3, 4) concern predictors of depression, two (Clusters 3, 5) the moderators, and the last two (Clusters 6, 8) the consequences of depression, as shown in Figure 3.

### Thematic Interpretation

#### Psychosocial, Affective–Cognitive and Lifestyle Predictors of Depression

Psychosocial factors, affective–cognitive factors, psychological stressors, and lifestyle factors are the primary predictors of depression identified in numerous studies. Collectively, these clusters reflect the substantial psychological burden experienced by medical students. Themes such as academic stress, social support, gender, and personality traits highlight the multifaceted nature of psychosocial vulnerabilities. The high co-occurrence of these terms suggests an interconnected framework in which stressors like inadequate social support and academic pressure amplify individual vulnerabilities, including gender-based differences and personality dispositions.

Within the affective–cognitive domain, keywords such as attitude, depression, emotional regulation, empathy, insomnia, and knowledge underscore the central role of internal psychological functioning, which is central to understanding mental health vulnerabilities in the medical student population. These findings emphasise that depression is not just an outcome of external stressors but is deeply influenced by internal processes like emotions, sleep cycles, and overall psychological functioning (19, 20). Although empathy is essential in a clinical and training setting, low levels or a lack of empathy combined with emotional exhaustion contribute to psychological distress (21). Moreover, sleep disturbances, especially insomnia, are highly prevalent among medical students and function as both a symptom and a contributing factor of depression symptomatology.

#### Psychosocial Stressors and Technology-Related Vulnerabilities

The high co-occurrence of terms such as anxiety, stress, COVID-19, pandemic, internet addiction, and psychological distress reflects the profound psychological burden experienced by medical students in recent years. The prominence of pandemic-related keywords highlights the cumulative impact of crisis-related stressors on an already vulnerable population. In parallel, the growing concerns over internet addiction, especially as a maladaptive coping mechanism, warrant further investigation due to its association with depression symptoms and poor academic outcomes (22, 23).

Lifestyle-related factors further complement these predictive domains. Limited physical activity and low quality of life were closely associated with psychological distress, indicating that the presence or absence of health-promoting behaviours is crucial to the well-being of medical students. Evidence suggests that the lack of an active lifestyle, often reinforced by academic overload and time constraints, significantly reduces quality of life and increases the risk of depression among medical students (24).

#### The Moderating Role of Coping Mechanisms and Burnout

The influence of these predictive factors is further shaped by key moderating variables such as coping mechanisms (Cluster 3) and Burnout (Cluster 5). Medical students employ a range of adaptive and maladaptive coping strategies in the high-pressure environment of medical education. The occurrence of coping and resilience indicates the importance of internal strength and behavioural strategies that may buffer the adverse effects of chronic academic and emotional stress (25).

However, several studies have underscored that higher perceived stress levels are associated with dysfunctional coping styles, including substance use and behavioural disengagement (26). In contrast, resilience functions as a protective factor that enables students to regulate their emotions despite persistent stressors (27). At the same time, however, maladaptive behaviours like smartphone addictions are increasingly prevalent and represent avoidant coping strategies that offer temporary distraction but exacerbate depression over time, as well as impairing the academic performance of students (28). Disturbed sleep quality is also linked to coping styles and contributes to elevated depression symptoms, particularly among students struggling with demanding academic schedules and emotional exhaustion (29).

Burnout represents a critical moderator within this framework. Chronic academic stress frequently results in burnout, which is characterised by emotional exhaustion, depersonalisation, and a reduced sense of personal accomplishment (30). Burnout is often reported to be prevalent among medical students due to the demanding nature of medical training. It often occurs with depressive symptoms, significantly impairing clinical performance and empathy levels (31). In severe cases, the combined effects of burnout and depression may lead to suicidal ideation and suicide attempts, which are disturbingly much more prevalent among medical students than in the general population (4).

#### Mental Health and the Academic Consequences of Depression

Within the proposed framework, depression is the primary outcome resulting from the interaction of multiple predictors and moderators. Persistent academic pressure and high performance demands place medical students at a heightened risk of a range of mental health problems (3). Evidence indicates that students who experience poor mental health are at increased risk of developing depression, experiencing burnout, and demonstrating declines in academic performance (32). This cluster emphasises the importance of identifying risk factors, both individual ones such as perfectionism, low self-esteem, and emotional dysregulation, and systemic factors, including lack of support and competitive environment, which contribute to the onset and maintenance of depressive symptoms (33).

The promotion of psychological wellbeing through resilience training, mindfulness practices, and improved mental health literacy has emerged as a critical direction of prevention and support. Poor academic performance represents a downstream consequence of depression. Psychological distress negatively affects academic outcomes, leading to lower grade point averages, increased absenteeism, and higher dropout rates (24). These findings underscore the need to conceptualise depression not only as an individual mental health issue but also as a systemic challenge that threatens the sustainability of the future healthcare workforce.

#### Integration into the Unified Conceptual Model

By integrating predictors, moderators, and consequences into a unified conceptual model, this framework provides a comprehensive understanding of depression among medical students that is expected to support the development of targeted, multi-level interventions aimed at reducing both the prevalence and the impact of depression within this population. While many studies employed cross-sectional designs, further longitudinal and qualitative research is needed to explore culturally contextualised risk factors and better capture the dynamic processes underlying depression among medical students.

### Future Research Directions

Our keyword analysis identified eight crucial thematic domains that represent potential avenues for future research on the topic of depression among medical students (RQ3). These themes provide a structured roadmap for advancing theoretical, empirical, and applied research areas.

#### Psychosocial Factors

Cluster 1 highlights the psychosocial factors contributing to depression among medical students. The most frequent terms within this cluster are medical students (occurrences = 225), depressive symptoms (occurrences = 28), and suicidal ideation (occurrences = 12). To achieve a deeper understanding of these relationships, future studies should explore causal pathways and mediating mechanisms, such as how personality dispositions may moderate the impact of academic stress on depressive outcomes. Furthermore, culturally grounded approaches and longitudinal studies are needed to clarify how gender roles and support systems shape mental health trajectories among medical students across diverse contexts. Comparative cross-cultural studies would also be valuable in understanding how sociocultural norms influence the manifestation and impact of psychosocial risk factors among medical students.

#### Affective–Cognitive Processes

Cluster 2 reflects the pivotal role of psychological mechanisms in the onset and persistence of depression among medical students. Future research should prioritise an in-depth examination of difficulties in emotional regulation, particularly in relation to academic and clinical stressors. Maladaptive emotional regulation strategies, such as rumination and emotional suppression, have been linked to elevated depression among medical students (20). Longitudinal studies in particular are needed to clarify the temporal relationship between sleep disturbances and depression. In addition to the persistent stigma around mental health within the medical population in many Asian regions, future research should address the negative attitudes and gaps in knowledge related to depression. Enhancing mental health literacy may facilitate the early identification of depression and encourage help-seeking behaviours (34). These findings also point to the need for structured psycho-educational programmes within medical curricula. Ultimately, future studies should investigate how affective–cognitive traits function as mediators or moderators between medical training stressors and mental health outcomes.

#### Coping Mechanism

Cluster 3 highlights the coping strategies employed by medical students in response to depression, as reflected by keywords like coping, medical education, perceived stress, resilience, smartphone addiction, and sleep quality. Given the consistency of both protective (e.g., resilience) and risk-inducing (e.g., smartphone addiction, poor sleep) factors, future research should focus on the typologies of coping among medical students. Intervention studies aimed at strengthening adaptive coping strategies, such as mindfulness, time management, and emotional regulation, should be included in the medical curriculum to improve student well-being and reduce psychological distress.

#### Psychological Stressors

Cluster 4 includes numerous psychological stressors that significantly contribute to the development of depression among medical students. The persistence of anxiety and psychological distress highlights the need for longitudinal research on the cumulative psychological impact of medical education, particularly in high-pressure training environments (3, 7). The strong association with COVID-19 and pandemic-related stressors further underscores the importance of examining the long-term mental health consequences of crisis events and their role in exacerbation existing vulnerabilities (35, 36).

Future studies should investigate post-pandemic mental health trajectories in medical students, especially in low- and middle-income countries, and examine how digital behaviours interact with psychological vulnerabilities. Excessive online activity often coexists with anxiety, sleep disturbances, and poor emotion regulation. Future studies should explore its role as both a symptom of and contributor to psychological decline in medical education. In addition, targeted stress management interventions, including stress inoculation training and resilience-building programmes, might be critical in mitigating the depressive effects of academic and pandemic-related pressures (37, 38).

#### Burnout in Depression

Burnout, sleep, and suicide were the keywords in Cluster 5, underscoring a strong interconnection between sleep disturbances, emotional exhaustion, and suicidal ideation among medical students. This cluster highlights an urgent need for future research focused on sleep hygiene interventions, the early identification of burnout symptoms, and comprehensive psychological support systems for medical trainees to prevent severe mental health outcomes like suicide.

#### Mental Health and Well-Being

Cluster 6 highlights the determinants of mental health and well-being. Future research should examine how institutional culture, peer dynamics, and stigma interact with psychological and environmental risk factors. There is also a growing need to develop comprehensive wellness models that emphasise psychological well-being, not merely symptom reduction (4). Prospective cohort studies could help untangle the temporal sequence between exposure to medical school stressors and the development of mental health symptoms.

#### Lifestyle Factors

Cluster 7 highlights the association between lifestyle behaviours and depression, centred on physical activity and quality of life. Future studies should explore how structured physical activity interventions in medical curricula can buffer against depression and emotional fatigue. Additionally, researchers should examine the mediating and moderating roles of factors such as time management and social support in relation to mental health and physical activity. Cross-cultural studies are also warranted to clarify how lifestyle practices influence mental health outcomes among medical students in different regions.

#### Academic Performance Outcome

Cluster 8 concerns the relationship between academic performance and depression. Future longitudinal studies are needed to explore and clarify the causal pathways between depression and academic decline. Research should also prioritise the early identification of academic-related stressors that negatively affect mental health, enabling timely interventions and academic support.

## Conclusion

This study provides a comprehensive bibliometric and thematic analysis of 476 Scopus-indexed publications on depression among medical students. Rather than focusing on citation performance or author productivity, the primary contribution of this study lies in the conceptual mapping of dominant and emerging research themes and the identification of future research directions.

The analysis highlights that research on depression among medical students is strongly anchored in the interrelated psychological, academic, psychosocial, and systematic factors associated with it. Key thematic clusters include psychological distress, academic pressure, coping mechanisms, burnout, lifestyle factors, and academic performance. Collectively, the findings indicate that depression in medical students is a multidimensional phenomenon shaped by the interactions of academic pressure, affective–cognitive processes, maladaptive coping strategies, sleep disturbances, limited physical activity, and broader systematic and cultural influences.

Additionally, emerging themes such as resilience and mental health literacy indicate a growing shift toward understanding and preventive approaches within medical education, suggesting an increasing recognition of the importance of understanding the protective factors alongside addressing the risk factors of depression. Based on the integration of these themes, this study highlights the need for a multi-level, interdisciplinary approach to address depression among medical students. The findings further highlight the importance of moving beyond cross-sectional studies to longitudinal, qualitative, and culturally grounded studies to understand the contextual mechanisms and different pathways underlying depression across regions.

Overall, this study contributes to the synthesis of our conceptual understanding of depression among medical students and provides directions for future research and policies to improve the mental health of medical students.

### Limitations of the Study

Despite its contributions, the study also has certain limitations that should be borne in mind. First, the search strategy was restricted to title-based terms related to depression and medical students, which might not include all the relevant research within Scopus, as some studies might address depression as the main outcome in medical students without explicitly including the terms in the title, which are instead found in their abstract or keywords. Since this study analysed data retrieved from the Scopus database, it is recommended that future research compare results across other databases such as Web of Science, Google Scholar, ScienceDirect, and PubMed. Despite these limitations, this study offers valuable insights into the structure, trends, and future directions of this research domain.

Second, although Scopus provides comprehensive coverage of data, reliance on a single database limits the generalisation of findings and may fail to fully capture the multidisciplinary scope of research in this area. In addition, geographical bias is evident from the concentration of research in certain regions, potentially underrepresenting the challenges and perspectives of less-studied areas, especially low- and middle-income countries. Limiting the search to English-language publications also created a language bias. The use of only one researcher in the screening process also introduced selection bias and might have reduced the reliability of the criteria for inclusion. Finally, this study only analysed publications from the past 25 years, not the entire period available; future research might examine earlier publications and provide a longer-term overview of the trends in specific time periods.

Notwithstanding these limitations, this study offers valuable insights into the thematic priorities and future directions of research on depression among medical students. By shifting the focus from bibliometric performance indicators to conceptual understanding, this study provides a strong foundation for researchers, educators, and policymakers to design context-sensitive interventions to improve mental health and well-being within medical education.

## Figures and Tables

**Figure 1:**
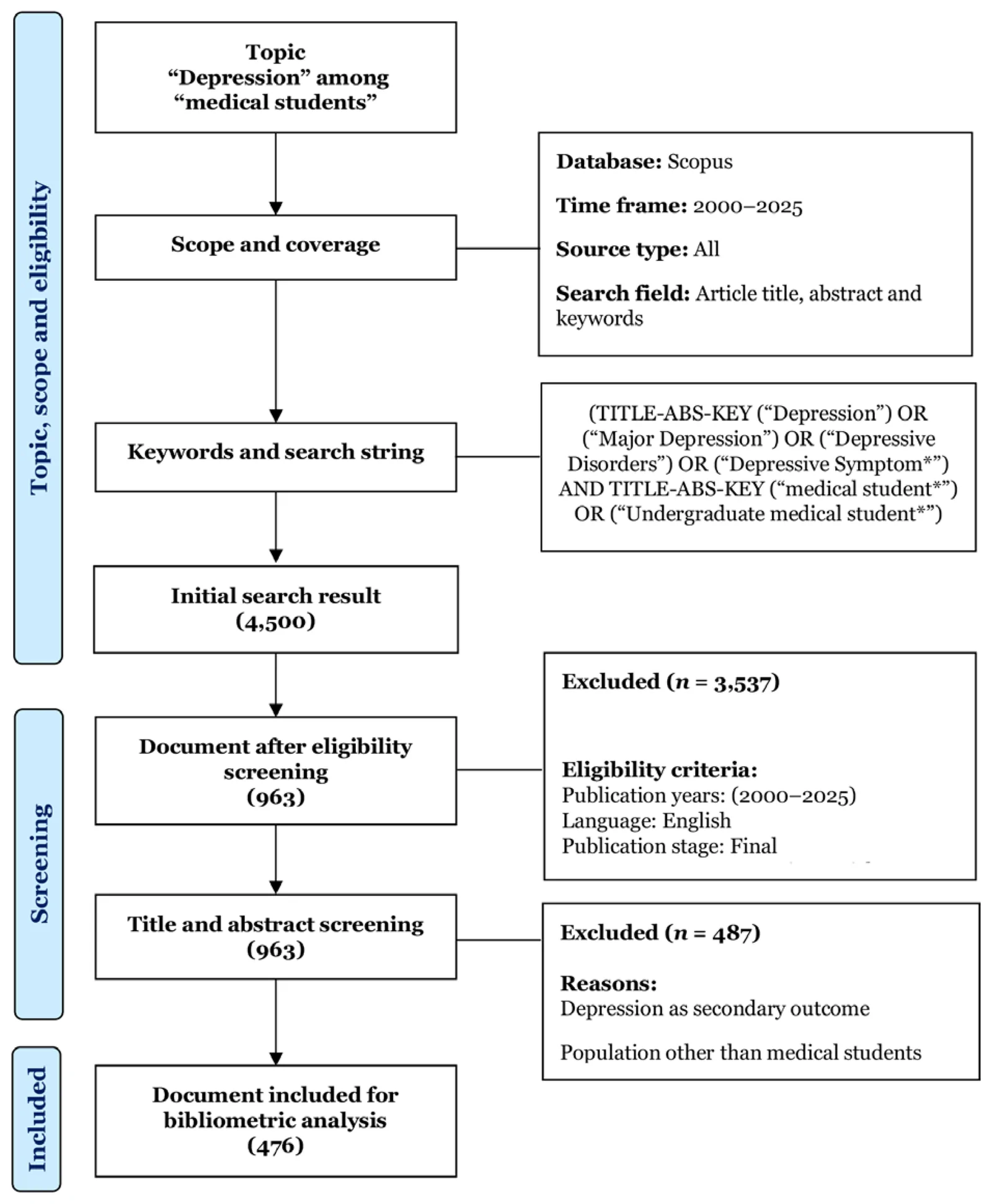
The flowchart of depression among medical students.

**Figure 2:**
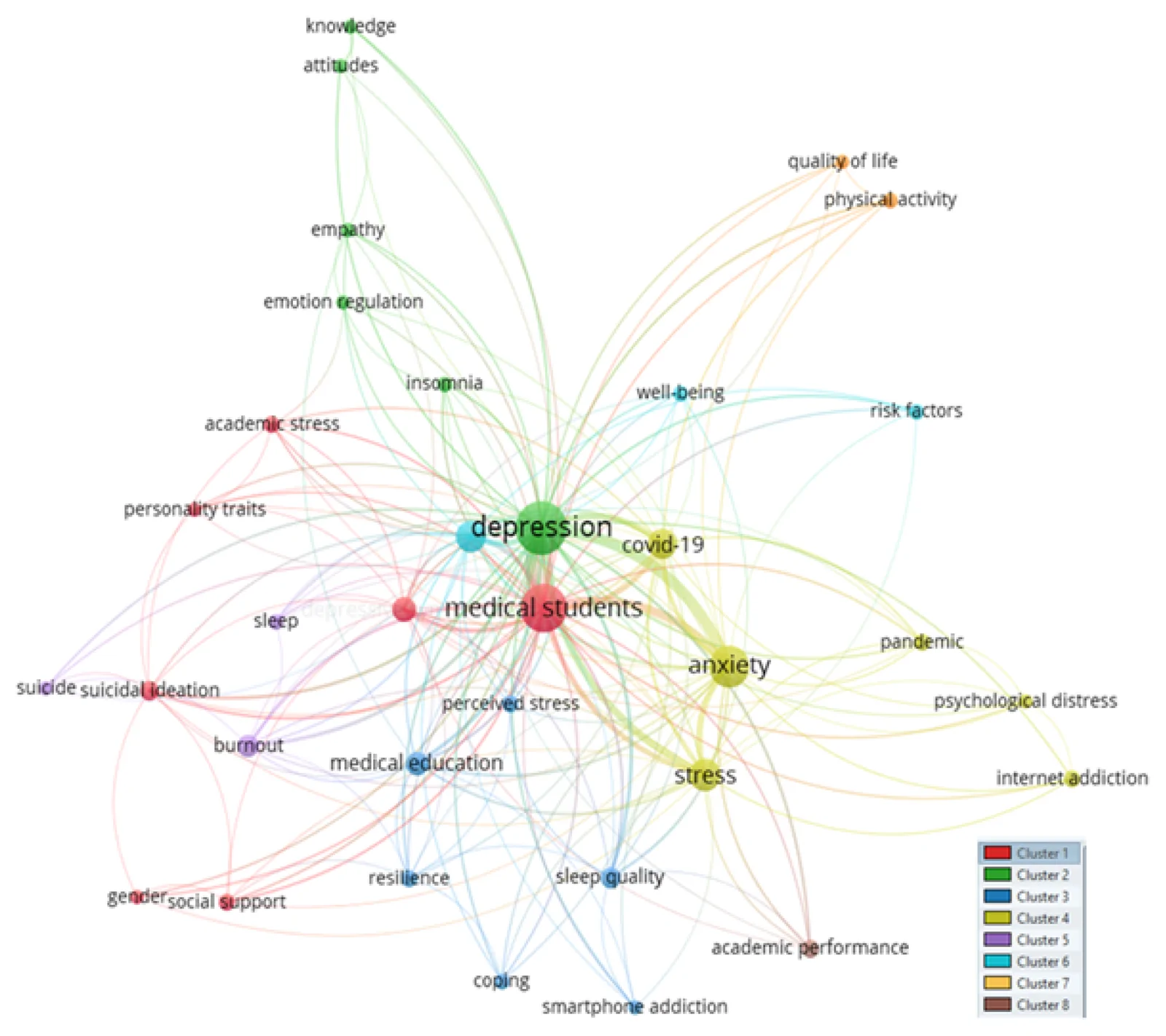
Network visualisation mapping with keyword co-occurrence.

**Figure 3:**
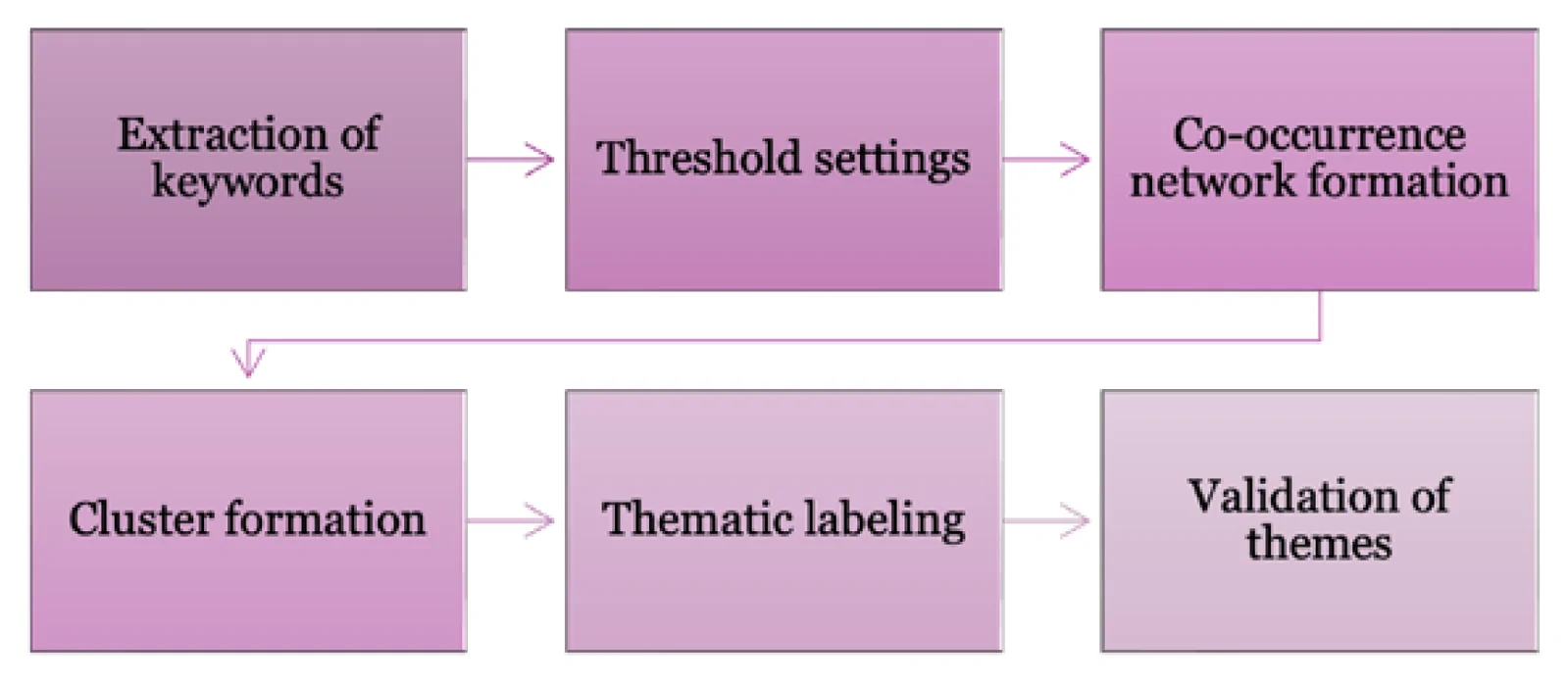
Process of thematic identification and cluster formation.

**Figure 4:**
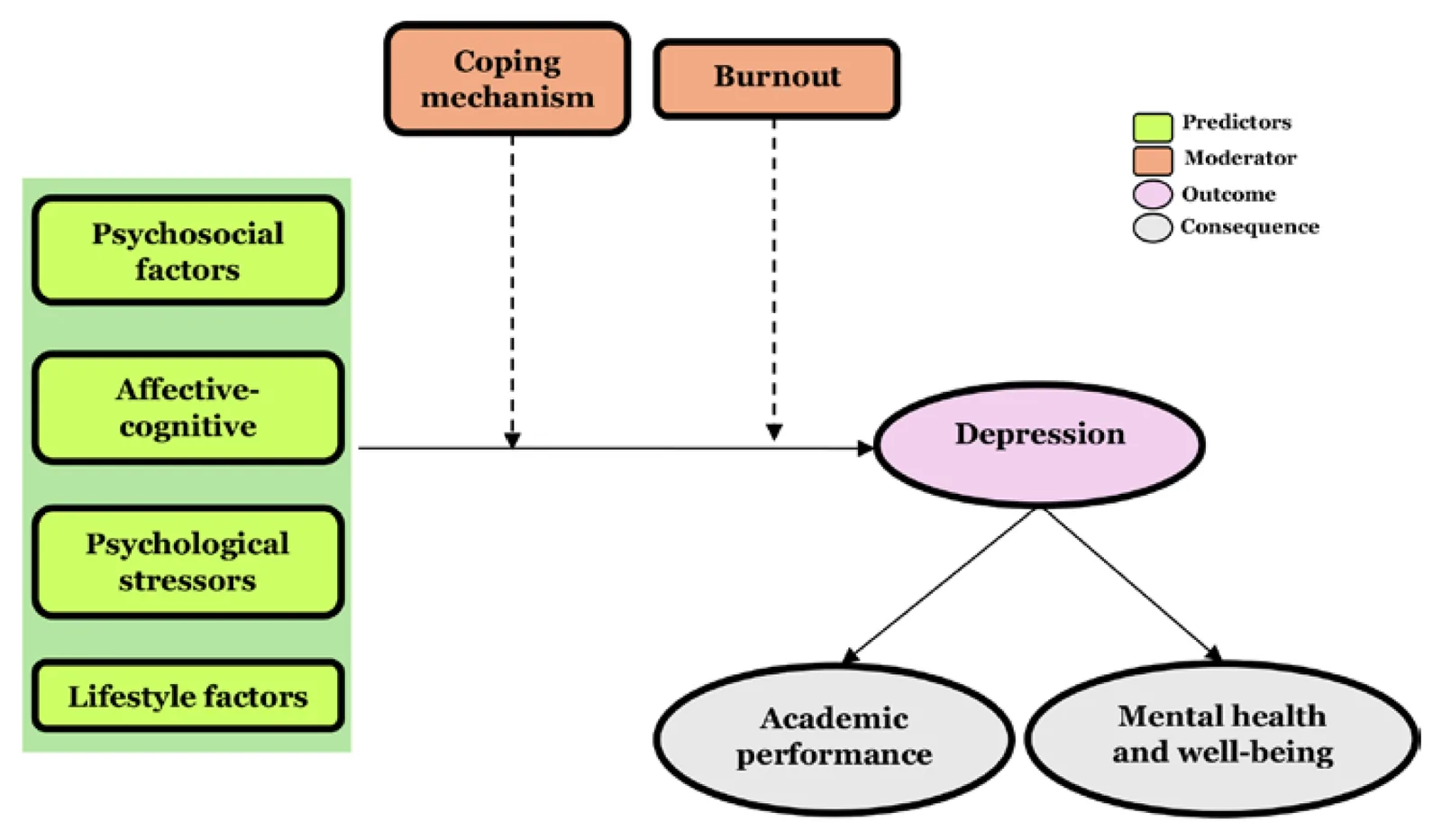
Conceptual framework based on eight clusters
